# Identification and genetic diversity analysis of *Rickettsia* in *Dermacentor nuttalli* within inner Mongolia, China

**DOI:** 10.1186/s13071-022-05387-4

**Published:** 2022-08-07

**Authors:** Zheng Gui, Hao Cai, Dong-Dong Qi, Shun Zhang, Shao-Yin Fu, Jing-Feng Yu, Xiao-Yan Si, Ting Cai, Rui Mao

**Affiliations:** 1 Key Laboratory of Diagnosis and Treatment of Digestive System Tumors of Zhejiang Province, Hwa Mei Hospital, University of Chinese Academy of Sciences, Ningbo, 315010 China; 2 Ningbo Institute of Life and Health Industry, University of Chinese Academy of Sciences, Ningbo, 315010 China; 3 Hulunbuir Mental Health Center, Hulunbuir, 022150 Inner Mongolia China; 4 Inner Mongolia Academy of Agricultural & Animal Husbandry Science, Hohhot, 010110 Inner Mongolia China; 5 Department of Parasitology, Inner Mongolia Medical University, Hohhot, 010110 Inner Mongolia China; 6 Inner Mongolia Center for Disease Control and Prevention, Hohhot, 010110 Inner Mongolia China

**Keywords:** *Rickettsia*, *gltA*, *ompA*, *Rickettsia* identification, Genetic diversity

## Abstract

**Background:**

The genus *Rickettsia* contains the lineages spotted fever group (SFG), typhus group (TG), and transitional group (TRG). The spotted fever group *Rickettsia* (SFGR) is transmitted by ticks. The tick species *Dermacentor nuttalli* is considered the main vector carrying SFGR in Inner Mongolia. Studying the genetic diversity and population structure of *Rickettsia* is essential for developing effective control strategies and predicting evolutionary trends of *Rickettsia*.

**Methods:**

In 2019 we collected 408 *D. nuttalli* in the Inner Mongolia Autonomous Region, detected the percentage of *Rickettsia*-positive specimens, and characterized the haplotypes. From the *Rickettsia*-positive ticks, the *gltA* and *ompA* genes were extracted, amplified, and sequenced.

**Results:**

Ten haplotypes of the *gltA* gene and 22 haplotypes of the *ompA* gene were obtained. The phylogenetic analysis showed that the haplotypes G1–G7 and G9 of the *gltA* gene cluster with *Rickettsia raoultii*, while G8 and G10 cluster with *Rickettsia sibirica*. Haplotypes O1–O15, O18 and O20–O22 of the *ompA* gene cluster with *R. raoultii*, while O16 and O19 cluster with *R. sibirica*. The average haplotype diversity was 0.3 for *gltA* and 0.7 for *ompA*. The average nucleotide diversity was greater than 0.05. Neutrality tests were nonsignificant for Tajima’s *D* results and Fu’s *F*s results. The fixation index values (*F*_ST_) showed that the degree of genetic differentiation between most sampled populations was small (*F*_ST_ < 0.05), whereas some populations showed a medium (*F*_ST_ > 0.05) or large (*F*_ST_ > 0.15) degree of differentiation. Analysis of molecular variance (AMOVA) revealed that the variation within populations was greater than that between populations. The mismatch analysis of *Rickettsia* showed double peaks.

**Conclusions:**

We found two *Rickettsia* spp. (*R. raoultii* and *R. sibirica*). The high genetic disparity of *Rickettsia* allows for easy adaption to different environments. Genetic differentiation between populations is small, and *Rickettsia* populations do not show a geographically differentiated structure. The high rates of retention and infection of *Rickettsia* in *D. nuttalli* together with the animal husbandry exchange in Inner Mongolia gradually led to the harmonization of genetic characteristics of *Rickettsia* across various regions. Overall, the significant genetic diversity and geographical structure of *Rickettsia* in *D. nuttalli* are critical for SFGR control.

**Graphical Abstract:**

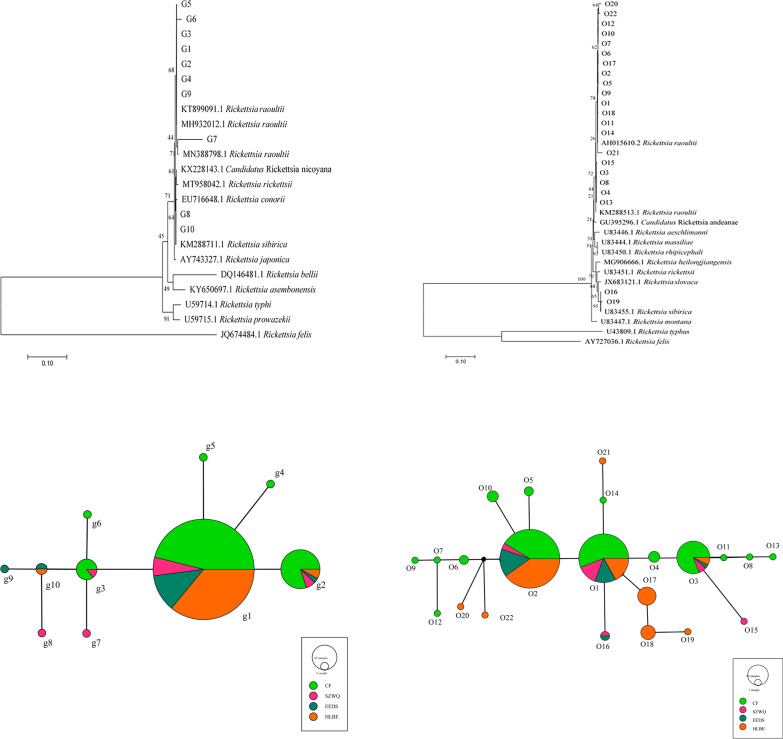

**Supplementary Information:**

The online version contains supplementary material available at 10.1186/s13071-022-05387-4.

## Background

Rickettsiosis is an important zoonosis, which brings serious harm to the health of humans and animals [1, 2]. The species of *Rickettsia* belong to three monophyletic groups: spotted fever group (SFG), typhus group (TG), and transitional group (TRG). SFG is transmitted by ticks [3, 4], while TG is transmitted by lice and fleas [5]. The vast territory and diverse habitats of Inner Mongolia greatly benefit the survival of ticks. *Dermacentor nuttalli* is the dominant tick species and is probably the main vector carrying spotted fever group *Rickettsia* (SFGR) in Inner Mongolia [6–8]. SFGR infections occur around the world and may cause serious diseases in humans. In China, *Rickettsia heilongjiangensis*, *Rickettsia raoultii*, *Rickettsia slovaca*, *Rickettsia sibirica*, *Rickettsia mongolotimonae*, *Rickettsia monacensis*, and *Candidatus* Rickettsia hebeiii and *Candidatus* Rickettsia jingxinensis have been detected in ticks. Furthermore, *Rickettsia raoultii*, *Rickettsia heilongjiangensis*, and *R. sibirica* have been reported in emerging tick-borne diseases of humans. In recent years, the threat that SFGR poses to public health in China has been magnified by the increasing number of potentially novel SFGR detected in ticks [9–11].

Molecular biological techniques allow for the identification of *Rickettsia* species. 16S rRNA can accurately identify a specimen as belonging to *Rickettsia*. However, it is difficult to distinguish the species, because the 16S rRNA sequence is highly conserved in almost all prokaryotes. The *gltA* and *ompA* genes are used for species identification in *Rickettsia* [12–14]. The *gltA* gene encodes a citrate synthase, the sequence of which allows for highly reliable identification of the evolutionary distances among *Rickettsia*. The *ompA* gene is an outer membrane protein gene. It is considered the “gold standard” for species identification in *Rickettsia*, owing to the highly specific 5′ end [15].

Previous studies found high genetic disparity in *D. nuttalli*, allowing for its existence in different geographical environments [16]. However, data on the genetic diversity of *Rickettsia* in *D. nuttalli* are scarce. Therefore, in our study, we included a high number of tick specimens, and explored the percentage of *Rickettsia*-positive samples and the genotype distribution to identify the genetic diversity of *Rickettsia*. Studying the genetic diversity of *Rickettsia* is essential for developing effective control strategies and predicting pathogen evolutionary trends.

## Methods

### Sample collection

**Fig. 1:**
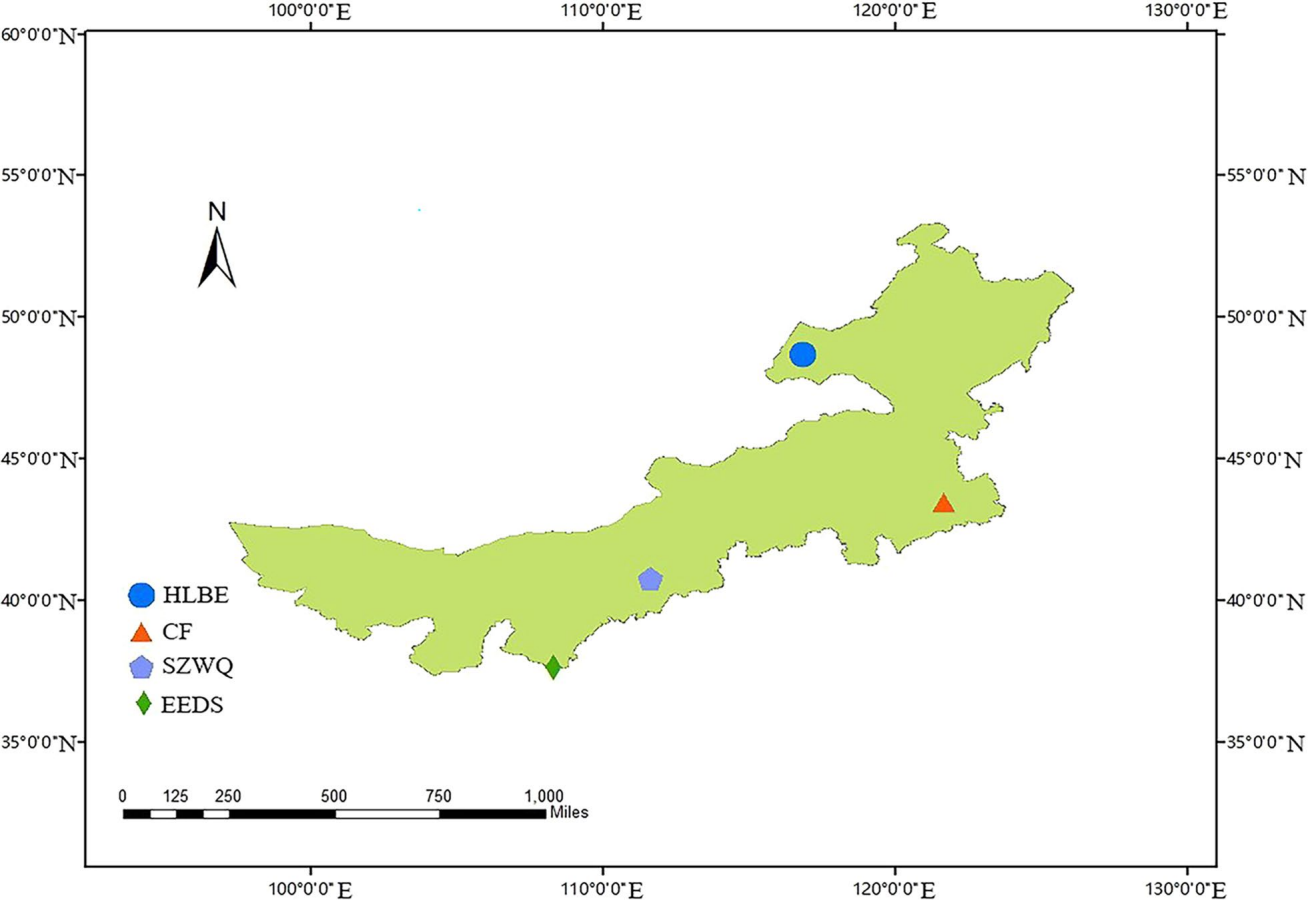
Collection site map. Samples of *D. nuttalli* were collected in four regions of Inner Mongolia. Colors indicate different collection regions in Inner Mongolia, and each graphic represents the approximate geographical coordinates of each collection site

In this study, a total of 408 ticks were collected from 1078 sheep at four sampling spots in Inner Mongolia, China: Chengchuan Town, early Banner of Etoke Banner Ordos (EEDS); Siziwang Banner, Hohhot (SZWQ); the Bayan WenduSumu area Arukorqin Banner Chifeng (CF); and Xinbarhu right Banner, Hulun Buir (HLBE). The locations of the sampling areas are shown in Additional file 1: Table S1 and Fig. 1.

### DNA extraction, amplification, and sequencing

Ticks were identified as *D. nuttalli* through morphological characteristics [17]. All *D. nuttalli* samples were individually extracted using a TIANamp Tissue and Blood Kit (TIANGEN, Beijing, China) [18]. For amplification of *Rickettsia* DNA, the *gltA* and *ompA* genes were amplified by polymerase chain reaction (PCR), following the protocols described by Bermúdez et al. [19]. Specific primers targeting the *gltA* and *ompA* genes of *Rickettsia* from *D. nuttalli* were synthesized by Shanghai Sangon. DNA was amplified using a system of 40 μl, each including Taq PCR Master Mix (Sangon, Shanghai, China), 2 μl of DNA from each sample, and 1 μl of each reverse and forward primer, and filled to volume with double-distilled water. The PCR primers, amplification sizes (base pairs), and annealing temperatures are listed in Additional file 1: Table S2. Double-distilled water was used as the negative control in each PCR reaction. Prior to sequencing, the quality of the PCR products was checked with 1.5% agarose gel electrophoresis stained with GoldView (Sangon, Shanghai, China). If the quality of the PCR product was suboptimal, it was purified using the Gel DNA Recovery Kit (TIANGEN, Beijing, China) and cloned using the pGEM-T Easy Vector System (Promega, Madison, WI, USA).

### Data analysis

Sequences were edited in SeqMan 7.1 and identified by comparative analysis with sequences deposited in GenBank, using the National Center for Biotechnology Information (NCBI) BLAST search engine. Sequencing data are available at the NCBI Sequence Read Archive (https://submit.ncbi.nlm.nih.gov/about/bankit/), with accession numbers OK638141-OK638150, OL304270-OL304271, OL348251-OL348270. Multiple sequence alignment and sequence similarity calculations were done using DNAMAN 7.0. Phylogenetic trees were constructed with the neighbor-joining algorithm using MEGA 7 with 1000 bootstrap replicates to assess tree stability [20–22]. Sequences were analyzed in DNAsp 5.10 and Arlequin 3.5 for calculating polymorphic sites, nucleotide differences, the number of haplotypes, both haplotype and nucleotide diversity, the distribution pattern of DNA haplotype variation, and genetic variation parameters [23, 24]. The extent of genetic differentiation among and between *Rickettsia* populations was estimated by analysis of molecular variance (AMOVA) and *F*_ST_ values [25]. To determine whether genetic differentiation and population structures of *Rickettsia* varied among the four sampling localities in Inner Mongolia. Neutral tests were analyzed using Tajima’s *D* and Fu’s *F*s tests using Arlequin 3.5 and DNAsp 5.10. PopART (Population Analysis with Reticulate Trees) version 1.7 software was used to evaluate the relationships between haplotypes by constructing TCS haplotype network maps.

## Results

### Detection of *Rickettsia* DNA

We collected and tested a total of 408 *D. nuttalli* in 2019 from four sites in Inner Mongolia: CF (*n* = 219), EEDS (*n* = 85), SZWQ (*n *= 30), and HLBE (*n* = 74) (Table 1). The quantity of amplification products of the *gltA* and *ompA* genes was regarded as the percentage of samples positive for *Rickettsia* in *D. nuttalli* [26]. Across the four regions, the average percentage of positive *Rickettsia* samples was 50.7%, with the highest value found in the HLBE region (85.1%).

**Table 1:** The percentage of *Rickettsia*-positive samples in *D. nuttalli*

Number	CF	EEDS	SZWQ	HLBE	Total
Tested	219	85	30	74	408
Positive	106	23	15	63	207
Positivity (%)	48.4	27.1	50.0	85.1	50.7

### *Rickettsia* identification

**Fig. 2:**
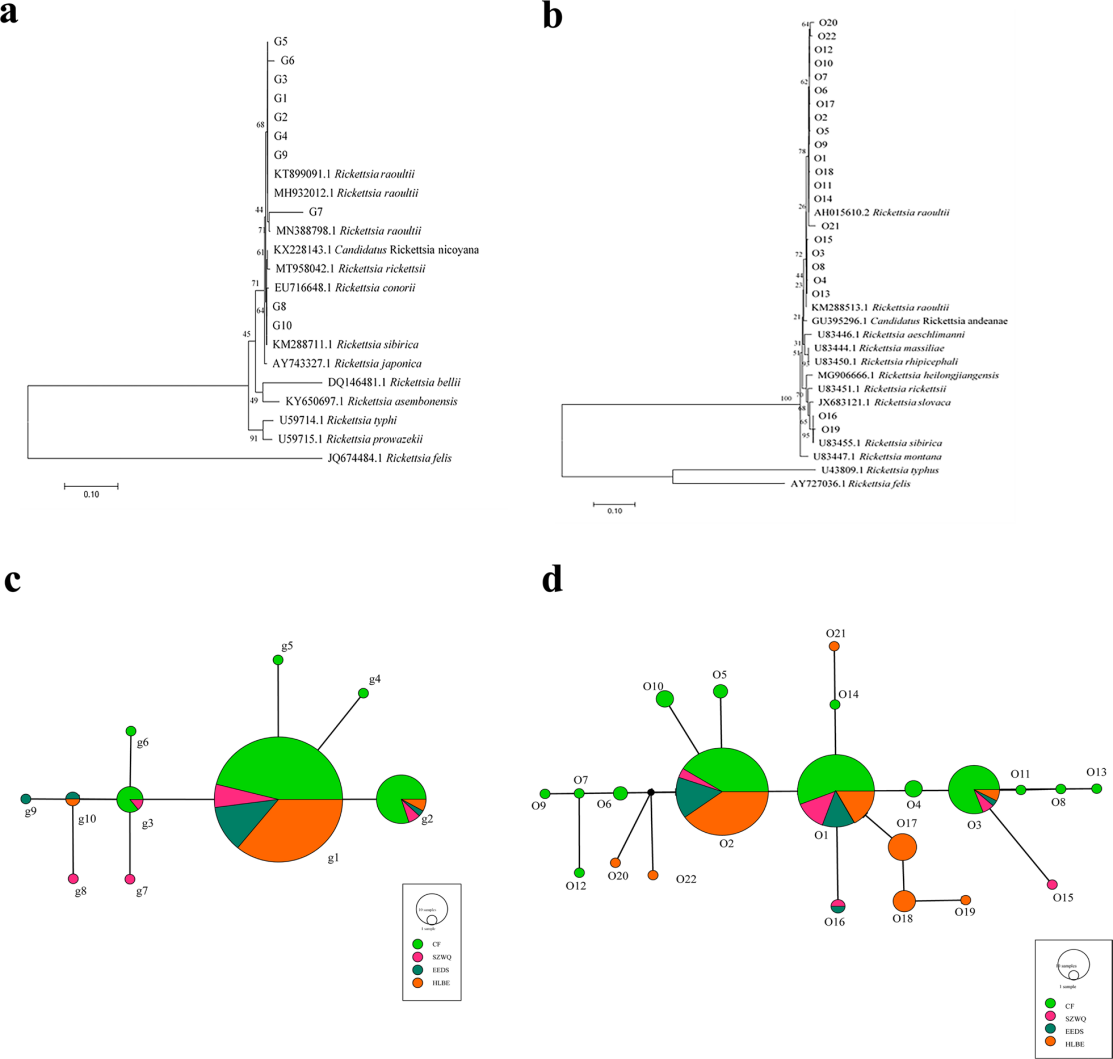
**a** Phylogenetic tree based on tbe *gltA* gene of *R. raoultii*. **b** Phylogenetic tree based on the *ompA* gene of *R. raoultii*. **c** TCS haplotype network of *R. raoultii* based on the *gltA* gene from four different populations in Inner Mongolia. **d** TCS haplotype network of *R. raoultii* based on the *ompA* gene from four different populations in Inner Mongolia

We detected 10 haplotypes of *gltA* sequences and 22 haplotypes of *ompA* sequences. The sequences had the highest similarity with *R. raoultii* and *R. sibirica*, as registered in GenBank, with 98% and 99% identity, respectively. In the *gltA* phylogenetic trees, haplotypes G1–G7 and G9 were clustered with *R. raoultii*, while G8 and G10 were clustered with *R. sibirica*. Furthermore, we found high similarity with *Candidatus* Rickettsia uralica, with 98% identity, and distinctly lower similarity in *Rickettsia asembonensis*. In the *ompA* phylogenetic trees, haplotypes O1–O15, O18, and O20–O22 clustered with *R. raoultii*, while O16 and O19 clustered with *R. sibirica*. The highest similarity was found with *Candidatus* R. uralica, with 98% identity, and substantially lower similarity with *Rickettsia montana*. The phylogenetic trees for both genes showed that the haplotypes of *Rickettsia* clustered into one branch with the ingroup, which contained the genotypes of *R. raoultii* and *R. sibirica* (Fig. 2a, b).

### *Rickettsia* genetic diversity by *gltA* gene

**Fig. 3:**
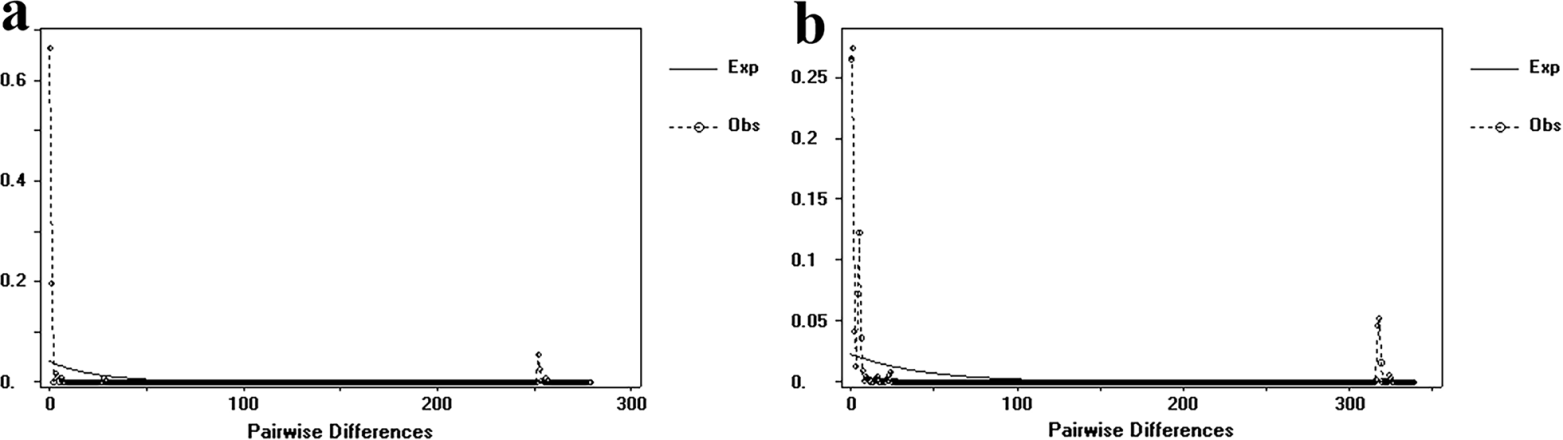
**a** Mismatch distribution analysis for the *R. raoultii* groups based on *gltA*. **b** Mismatch distribution analysis for the *R. raoultii* groups based on *ompA*

In the *gltA* sequences, the final alignment consisted of 1318 base pairs, with 842 variable sites. Of 10 haplotypes recovered, four were shared haplotypes (G1, G2, G3, G10). Numerically, the most common haplotype was G1, with 167 sequences (80.7% of all sequences) (Additional file 1: Table S3). G1 turned out to be the dominant haplotype. It was placed in the center of the haplotype network and was found in four geographically separate populations (Fig. 2c). The average haplotype diversity was 0.335. The average nucleotide diversity was 0.04922. EEDS was the region with the highest haplotype diversity (*h* = 0.909). Neutrality analysis revealed nonsignificant values of Tajima's *D* and Fu's *F*s results, confirming that the population had not experienced recent expansion (Table 2). Wright’s *F* index was calculated to measure the levels of genetic differentiation among the four geographical populations, indicating the allelic variation between populations, which correlated negatively with gene flow. Comparing pairwise *F*_ST_ indices showed that the *F*_ST_ value between HLBE and SZWQ was greater than 0.25, indicating high genetic differentiation among populations, probably on account of low gene flow. The *F*_ST_ values among the other regions were less than 0.05, indicating that the genetic differentiation among these populations was very small, with a high degree of gene flow (Additional file 1: Table S4). The AMOVA showed that the variability in *Rickettsia* mainly arose from within each population, and the genetic differentiation between populations was very small (Additional file 1: Table S5). The mismatch analysis presented double peaks, indicating that the four geographical populations did not experience rapid population expansion (Fig. 3a).

**Table 2:** Summary statistics for polymorphism and neutrality tests of the *gltA* gene from *Rickettsia* in Inner Mongolia

	No.	Ht	S	Hd	Pi	Tajima’s *D*	Fu’s *F*s
CF	106	14	400	0.484	0.06725	−1.47773	47.607
EEDS	23	12	445	0.909	0.04918	−2.72424**	8.007
HLBE	63	6	10	0.313	0.00064	−2.13550*	−2.666
SZWQ	15	9	308	0.800	0.13230	−1.12826	9.686
TOTAL	207	10	282	0.335	0.04922	−1.65779	46.426

Ht, number of haplotypes, *S* number of polymorphic sites, *Hd* haplotype diversity, *Pi* nucleotide diversity

*p < 0.05

**p < 0.01

***p < 0.001

### **Rickettsia** genetic diversity by *ompA* gene

In the *ompA* sequences, the final alignment consisted of 738 base pairs, with 466 variable sites. Of the 22 haplotypes that were found, four were shared haplotypes (O1, O2, O3, O16). Numerically, the most common haplotypes were O1, O2, and O3, with 170 sequences (82.1% of all sequences) (Additional file 1: Table S6). O2 was the dominant haplotype. It was placed in the center of the haplotype network and was found in four different populations (Fig. 2d). The total average haplotype diversity was 0.735 and the total nucleotide diversity was 0.07308. SZWQ was the locality with the highest haplotype diversity (*h* = 1). Neutrality results were the same as those for *gltA*, indicating that the population had not experienced expansion recently (Table 3). The *F*_ST_ value between CF and HLBE was greater than 0.25, indicating that there was high genetic differentiation between populations. The *F*_ST_ values between HLBE, SZWQ, and EEDS were all greater than 0.05, confirming a moderate genetic differentiation between populations, with a small extent of gene flow. The *F*_ST_ values between the other regions were less than 0.05, confirming a very small genetic differentiation between these populations, likely on account of high gene flow (Additional file 1: Table S7). The results of the AMOVA and mismatch analyses were consistent with the *gltA* results (Additional file 1: Table S8, Fig. 3b).

**Table 3:** Summary statistics for polymorphism and neutrality tests of the *ompA* gene from *Rickettsia* in Inner Mongolia

	No.	Ht	S	Hd	Pi	Tajima’s *D*	Fu’s *F*s
CF	106	15	11	0.765	0.00394	−0.26745	−3.216
EEDS	23	12	43	0.889	0.00947	−2.21147**	−1.104
HLBE	63	15	365	0.739	0.19544	1.53899	58.425
SZWQ	15	15	57	1	0.02468	−1.48940	−5.059
TOTAL	207	22	349	0.735	0.07308	−1.08797	48.645

*Ht* number of haplotypes, *S* number of polymorphic sites, *Hd* haplotype diversity, *Pi* nucleotide diversity

*p < 0.05

**p < 0.01

***p < 0.001

## Discussion

For planning effective control measures against *Rickettsia* infection, it is of utmost importance to continuously monitor the emergence of new species, study the population structure, and investigate the genetic diversity of the pathogen. In this study, two *Rickettsia* species were identified in Inner Mongolia, *R. raoultii* and *R. sibirica*, both belonging to the SFGR. In recent years, human infection with *R. raoultii* has led to tick-borne lymphadenitis in many countries [27–30]. To strengthen appropriate detection and treatment measures in endemic regions, public health workers and physicians should pay close attention to the high risk of human infection by *R. raoultii*.

This study analyzed the genetic diversity of a region within *gltA* and *ompA* genes of *Rickettsia* from four localities of Inner Mongolia. The aim of the investigation was to find new possibilities for controlling the transmission and reproduction of *Rickettsia*. Our results showed that the *gltA* and *ompA* genes have shared haplotypes in four regions. These are dominant haplotypes characterized as primitive and stable. Shared haplotypes indicate that the degree of genetic communication is high in *Rickettsia* populations. Furthermore, the lowest genetic diversity was found in HLBE in the *gltA* gene. The diversity of the *ompA* was higher in HLBE. The different results for the two genes indicate that the genetic diversity of species is affected by many factors, such as geographical distribution and population size [31]. Different gene markers are under different selective pressure during the evolution of species, leading to inconsistent genetic diversity [32]. Overall, the high genetic diversity of *Rickettsia* is in accord with different environments. The high rate of genetic communication in *Rickettsia* populations not only leads to a higher positive rate of *Rickettsia* in Inner Mongolia, but also increases the probability of *Rickettsia* transmission among and between humans and livestock.

Regarding the four geographical populations investigated, there is genetic differentiation between populations from AMOVA results. Further studies on *F*_ST_ values revealed that the degree of genetic differentiation was highest between HLBE and the other three regions. Obviously, HLBE is not only a beneficial habitat for ticks but also an important pastoral region in Inner Mongolia [33]. It also has the highest *Rickettsia*-positive rate and the highest genetic differentiation in *Rickettsia* populations in Inner Mongolia.

The neutrality test values of Tajima’s *D* and Fu’s *F*s are used to test the historical dynamics of populations. If both are significantly negative, it indicates that the *D. nuttalli* population has historically experienced rapid population expansion. In this study, we did not find evidence of a recent rapid expansion of the population. Furthermore, the mismatch analysis for the two genes *gltA* and *ompA* showed genetic differentiation with no population expansion. The haplotypes of *Rickettsia* did not branch in relation to the clustering of geographical regions, indicating that the *Rickettsia* populations did not form a geographical differentiation structure. Therefore, in recent years, *Rickettsia* populations have not experienced excessive outbreaks, and the phenomenon of high genetic diversity may be a historical effect.

Since *Rickettsia* can be transmitted either vertically or horizontally, the high carry and infection rates of *Rickettsia* in *D. nuttalli*, together with the animal husbandry exchange in Inner Mongolia, gradually led to the harmonization of genetic characteristics of *Rickettsia* across various regions. The National Center for Disease Control and Prevention should strengthen the monitoring of tick-borne *Rickettsia* in Inner Mongolia and develop effective control measures.

## Conclusion

The *gltA* and *ompA* genes were used to study the genetic diversity of *Rickettsia* from four geographical localities in Inner Mongolia. This study provides a reference for detecting new genotypes and complex genetic structures of *Rickettsia* populations. It also indicates that although *Rickettsia* species in Inner Mongolia have adapted to different environments, effective control measures of tick-borne *Rickettsia* transmission is crucial, especially in the HLBE region.

## Supplementary Information

**Additional file 1: Table S1.** Sample information of *D. nuttalli* populations. **Table S2.** Primers of PCR and amplification conditions**. Table S3** Haplotype distribution of *Rickettsia* based on the *gltA* gene. **Table S4.** *F*_ST_ values among different groups of *Rickettsia* based on the *gltA* gene. **Table S5.** AMOVA of *gltA* gene of *Rickettsia* population. **Table S6** Haplotype distribution of *Rickettsia* based on the *ompA* gene. **Table S7** *F*_ST_ values among different groups of *Rickettsia* based on the *ompA* gene. **Table S8** AMOVA of the *ompA* gene of the *Rickettsia* population.

## Data Availability

All datasets have been included with this article and our sequences have been deposited within GenBank (accession number OK638141-OK638150 for *gltA*, accession number OL304270-OL304271, OL348251-OL348270 for *ompA*).

## Abbreviations

PCR: Polymerase chain reaction
*D. nuttalli*: *Dermacentor nuttalli*
*R. raoultii*: *Rickettsia raoultii*
*R. sibirica*: *Rickettsia sibirica*
AMOVA: Analysis of molecular variance
BLAST: Basic Local Alignment Search Tool
