# Supplementary material for: Identification and genetic diversity analysis of Rickettsia in Dermacentor nuttalli within inner Mongolia, China
Source: Parasit Vectors. 2022 Aug 7;15:286. doi: 10.1186/s13071-022-05387-4 (PMC9358909; doi:10.1186/s13071-022-05387-4)
Supplement: Supplementary file 1 — Additional file 1: Table S1. Sample information of D. nuttalli populations. Table S2. Primers of PCR and amplification conditions. Table S3 Haplotype distribution of Rickettsia based on the gltA gene. Table S4. FST values among different groups of Rickettsia based on the gltA gene. Table S5. AMOVA of gltA gene of Rickettsia population. Table S6 Haplotype distribution of Rickettsia based on the ompA gene. Table S7 FST values among different groups of Rickettsia based on the ompA gene. Table S8 AMOVA of the ompA gene of the Rickettsia population. [file 13071_2022_5387_MOESM1_ESM.docx]

**Identification and genetic diversity analysis of *Rickettsia* in *Dermacentor nuttalli* within Inner Mongolia, China**

Zheng Gui^1, 2+^, Hao Cai^1, 2+^, Dong-Dong Qi^3+^, Shun Zhang^1, 2^, Shao-Yin Fu^4^, Jing-Feng Yu^5*^, Xiao-Yan Si^6*^, Ting Cai^1, 2*^ and Rui Mao^1, 2*^

+Equal contributors

^1^Key Laboratory of Diagnosis and Treatment of Digestive System Tumors of Zhejiang Province, Hwa Mei Hospital, University of Chinese Academy of Sciences, Ningbo 315010, China.

^2^Ningbo Institute of Life and Health Industry, University of Chinese Academy of Sciences, Ningbo 315010, China.

^3^ Hulunbuir Mental Health Center, Hulunbuir 022150, Inner Mongolia, China.

^4^ Inner Mongolia Academy of Agricultural & Animal Husbandry Science, Inner Mongolia 010110, China.

^5^Department of Parasitology, Inner Mongolia Medical University, Inner Mongolia 010110, China.

^6^ Inner Mongolia Center for Disease Control and Prevention, Inner Mongolia 010110, China.

^*^Correspondence: 1184474898@126.com, 372304169@qq.com, caiting@ucas.ac.cn, maorui@ucas.ac.cn

E-mails:

ZG: 15248097967@sina.cn

HC: 960243644@qq.com

DDQ: 1420352185@qq.com

SZ: zhangshun@ucas.ac.cn

SYF: fushao1234@126.com

JFY: 1184474898@126.com

XYS: 372304169@qq.com

TC: caiting@ucas.ac.cn

RM: maorui@ucas.ac.cn

**Supplement Table S1**

**Table S1** Sample information of *D. nuttalli* populations

| Location | Collection time | Area code | Longitude | Latitude | Samples | Host (Sheep) |
| --- | --- | --- | --- | --- | --- | --- |
| ChiFeng | 2019.4 | CF | 121°64′ | 43°46′ | 219 | 570 |
| Siziwang Banner | 2019.5 | SZWQ | 111°63′ | 40°81′ | 30 | 73 |
| Hulun Buir | 2019.8 | HLBE | 116°82′ | 48°67′ | 74 | 180 |
| Ordos | 2019.7 | EEDS | 108°32′ | 37°70′ | 85 | 255 |

**Supplement Table S2**

**Table S2** Primers of PCR and amplification conditions

| Gene target | Primer sequence（5'**~**3'） | Amplification size（bp） | Annealing temperature（°C） |
| --- | --- | --- | --- |
| *gltA* | F: CGATTGCTTTACTTACGACCC | 1090 | 53 |
|  | R: GAGCATTTCTTTCCATTGTGCC |  |  |
| *ompA* | F: ACTAGGTGCGAATATAGACCC | 712 | 56 |
|  | R: CGTACCTTTTGCGTTAACACT |  |  |

**Supplement Table S3**

**Table S3** Haplotype distribution of *Rickettsia* based on *gltA* gene

| Hap | Num | CF | EEDS | HLBE | SZWQ |
| --- | --- | --- | --- | --- | --- |
| G1  G2  G3  G4  G5  G6  G7  G8  G9  G10 | 167  25  7  1  1  1  1  1  1  2 | 77  20  6  1  1  1  0  0  0  0 | 20  1  0  0  0  0  0  0  1  1 | 60  2  0  0  0  0  0  0  0  1 | 10  2  1  0  0  0  1  1  0  0 |

**Supplement Table S4**

**Table S4** F_ST_ values among different groups of *Rickettsia* based on *gltA* gene

| Locality | CF | EEDS | HLBE | SZWQ |
| --- | --- | --- | --- | --- |
| CF |  |  |  |  |
| EEDS | -0.02141 |  |  |  |
| HLBE | 0.04136 | 0.04878 |  |  |
| SZWQ | -0.00537 | -0.00118 | 0.25240 |  |

**Supplement Table S5**

**Table S5** AMOVA of *gltA* gene of *Rickettsia* population

| Source of variation | Degrees of freedom | | Sum of squares | Variance Components | Percentage of variation | Fixation Idex FST |
| --- | --- | --- | --- | --- | --- | --- |
| Among population  Within population  Total | | 3  203  206 | 75.094  294.027  2469.121 | 0.30572Va  11.79324Vb  12.09896 | 2.52684  97.47316 | 0.02527 |

**Supplement Table S6**

**Table S6** Haplotype distribution of *Rickettsia* based on *ompA* gene

| Hap | Num | CF | EEDS | HLBE | SZWQ |
| --- | --- | --- | --- | --- | --- |
| O1  O2  O3  O4  O5  O6  O7  O8  O9  O10  O11  O12  O13  O14  O15  O16  O17  O18  O19  O20  O21  O22 | 59  85  26  3  2  2  1  1  1  3  1  1  1  1  1  1  8  5  1  1  1  1 | 33  35  21  3  2  2  1  1  1  3  1  1  1  1  0  0  0  0  0  0  0  0 | 8  13  1  0  0  0  0  0  0  0  0  0  0  0  0  1  0  0  0  0  0  0 | 10  34  2  0  0  0  0  0  0  0  0  0  0  0  0  0  8  5  1  1  1  1 | 8  3  2  0  0  0  0  0  0  0  0  0  0  0  1  1  0  0  0  0  0  0 |

**Supplement Table S7**

**Table S7** F_ST_ values among different groups of *Rickettsia* based on *ompA* gene

| Locality | CF | EEDS | HLBE | SZWQ |
| --- | --- | --- | --- | --- |
| CF |  |  |  |  |
| EEDS | 0.04464 |  |  |  |
| HLBE | 0.25852 | 0.13410 |  |  |
| SZWQ | 0.01902 | 0.01996 | 0.11367 |  |

**Supplement Table S8**

**Table S8** AMOVA of *ompA* gene of *Rickettsia* population

| Source of variation | | d.f | Sum of squares | Variance Components | Percentage of variation | Fixation  Idex FST |
| --- | --- | --- | --- | --- | --- | --- |
| Among population  Within population  Total | 3  203  206 | | 691.719  3696.644  4388.362 | 4.90433Va  18.21007Vb  23.11439 | 21.21763  78.78237 | 0.21218 |
